# Structure and function of mitochondrial membrane protein complexes

**DOI:** 10.1186/s12915-015-0201-x

**Published:** 2015-10-29

**Authors:** Werner Kühlbrandt

**Affiliations:** Department of Structural Biology, Max Planck Institute of Biophysics, Max von Laue Str. 3, D-60438 Frankfurt am Main, Germany

## Abstract

Biological energy conversion in mitochondria is carried out by the membrane protein complexes of the respiratory chain and the mitochondrial ATP synthase in the inner membrane cristae. Recent advances in electron cryomicroscopy have made possible new insights into the structural and functional arrangement of these complexes in the membrane, and how they change with age. This review places these advances in the context of what is already known, and discusses the fundamental questions that remain open but can now be approached.

**Electronic supplementary material:**

The online version of this article (doi:10.1186/s12915-015-0201-x) contains supplementary material, which is available to authorized users.

Mitochondria are the powerhouses of the cell. In all eukaryotes that do not depend on photosynthesis, the mitochondria are the main source of adenosine triphosphate (ATP), the energy-rich compound that drives fundamental cell functions. These functions include force generation (for example, in muscle contraction and cell division), the biosynthesis, folding and degradation of proteins, and the generation and maintenance of membrane potentials. ATP is produced on a massive scale in the human body, amounting to 50 kg per day in a healthy adult, but considerably more in a long-distance runner. ATP is generated by the mitochondrial ATP synthase from ADP and phosphate ions. These are the products of ATP hydrolysis at the sites where energy is needed in the cell. Apart from cellular respiration and ATP synthesis, mitochondria have numerous other essential functions, including the production of NADH and GTP in the citric acid cycle, the biosynthesis of amino acids, heme groups and iron-sulfur clusters or the synthesis of phospholipids for membrane biogenesis. They also act in calcium signaling [1], stress responses [2] and generally as cellular signaling hubs [3]. Not surprisingly, mitochondria play a fundamental role in human health. Mitochondrial dysfunction is the cause of severe, often maternally inherited diseases. Moreover, mitochondria are deeply implicated in apoptosis and ageing [4].

In many respects, mitochondria resemble α-proteobacteria, from which they are thought to have originated by endocytosis some 1.6 billion years ago. The most striking evidence of this evolutionary relationship is the close homology of bacterial and mitochondrial respiratory chain complexes. Mitochondria have their own genetic system, which uses a distinct DNA code that differs both from that of their bacterial ancestors and their eukaryotic hosts [5]. They have their own protein translation machinery, complete with ribosomes, tRNAs and associated protein factors that more or less resemble those of their bacterial ancestors. Very recently, the first high-resolution structure of a mitochondrial ribosome, determined by single-particle electron cryomicroscopy (cryo-EM), has revealed a fascinating patchwork of similarities to and differences from bacterial ribosomes [6]. Nevertheless, mitochondria make surprisingly little use of their specialized protein production machinery. In the course of evolution they have transferred up to 99 % of their genes to the nucleus. Today, the vast majority of mitochondrial proteins are produced in the cytoplasm and imported into the organelle by an elaborate set of protein translocases [7]. In humans, only 13 mitochondrial proteins are organelle-encoded, all of them central, hydrophobic subunits of respiratory chain complexes or of the ATP synthase.

Mitochondria are highly dynamic [8]. In the cell, they form a tubular network that constantly changes by division and fusion (Additional file 1). Both processes are accomplished by multi-component molecular machineries that include a number of dynamin-related GTPases [9, 10]. When mitochondria are isolated from cells, the network breaks up into fragments that spontaneously reseal. Isolated mitochondria are fully competent for respiration and ATP synthesis [11]. They maintain their membrane composition, organization and membrane potential, as well as the ability to fuse [12] and to import proteins [7]. We owe much of what we know about mitochondria and how they work at the molecular level to in vitro studies with isolated mitochondria, or even mitochondrial membrane fractions, which still carry out oxidative phosphorylation and ATP synthesis [13].

Mitochondria can be seen in the light microscope, but their detailed internal structure is only revealed by electron microscopy. In the 1990s, the structure of mitochondria was investigated by electron tomography of thin plastic sections [14]. While this yielded striking three-dimensional (3D) images of their internal membrane system, molecular detail was lost due to chemical fixation, dehydration and heavy-metal staining. Cryo-EM of unfixed, unstained organelles is now revealing the architecture of mitochondrial membranes and their macromolecular components at increasing levels of detail. Single-particle cryo-EM of isolated, detergent-solubilized membrane protein complexes reaches near-atomic resolution [15, 16]. Electron cryo-tomography (cryo-ET) of intact isolated mitochondria or mitochondrial membranes is resolving their macromolecular components in situ [17], and averaging of tomographic volumes can attain sub-nanometer resolution [18].

## Mitochondrial membranes and membrane compartments

**Fig. 1:**
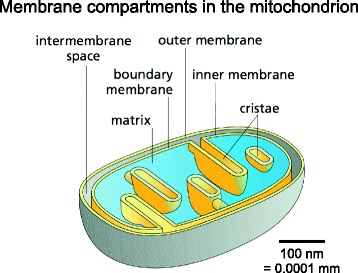
Membrane compartments in the mitochondrion. The outer membrane separates mitochondria from the cytoplasm. It surrounds the inner membrane, which separates the inter-membrane space from the protein-dense central matrix. The inner membrane is differentiated into the inner boundary membrane and the cristae. The two regions are continuous at the crista junctions. The cristae extend more or less deeply into the matrix and are the main sites of mitochondrial energy conversion. The shallow proton gradient between the inter-membrane space (pH 7.2–7.4) and the matrix (pH 7.9–8) drives ATP production by the ATP synthase in the membranes of the cristae. (Adapted from Figure 14–8 C in Alberts B. et al. Molecular Biology of the Cell. 6^th^ ed. New York: Garland Science; 2014, with permission of the publisher [© 2015 from Molecular Biology of the Cell, Sixth Edition by Alberts et al. Reproduced by permission of Garland Science/Taylor & Francis Group LLC])

As ubiquitous, semi-autonomous cellular organelles, mitochondria are separated from the cytoplasm by the outer and inner mitochondrial membrane (Fig. 1). The outer membrane is porous and freely traversed by ions and small, uncharged molecules through pore-forming membrane proteins (porins), such as the voltage-dependent anion channel VDAC [19]. Any larger molecules, especially proteins, have to be imported by special translocases. Because of its porosity, there is no membrane potential across the outer membrane. By contrast, the inner membrane is a tight diffusion barrier to all ions and molecules. These can only get across with the aid of specific membrane transport proteins, each of which is selective for a particular ion or molecule. As a result of its ion selectivity, an electrochemical membrane potential of about 180 mV builds up across the inner mitochondrial membrane. The inner membrane is where oxidative phosphorylation takes place in a suite of membrane protein complexes that create the electrochemical gradient across the inner membrane, or use it for ATP synthesis.

The inner and outer membranes of mitochondria define three compartments within the organelle, each with its distinct role and corresponding protein components. The innermost compartment, surrounded by the inner membrane, is the mitochondrial matrix. It is the equivalent of the bacterial cytoplasm, from which it is distinguished by a pH of 7.9 to 8 [20], similar to that in the chloroplast stroma. The high pH of the mitochondrial matrix creates the trans-membrane electrochemical gradient that drives ATP synthesis (see below). The mitochondrial matrix is the site of organellar DNA replication, transcription, protein biosynthesis and numerous enzymatic reactions. Mitochondrial DNA is compacted by the mitochondrial transcription factor TFAM into supramolecular assemblies called nucleoids, of which there are about 1000 per cell [21]. Nucleoids are roughly spherical, with a diameter of ~100 nm, each containing one copy of mitochondrial DNA [22]. Mitochondrial ribosomes are membrane-attached, as their only products (in human cells) are hydrophobic membrane protein subunits, which integrate directly into the inner membrane upon translation. A ~25 Å resolution structure of the membrane-bound mitochondrial ribosome has recently been obtained by cryo-ET and sub-tomogram averaging [23].

The biosynthetic reactions that happen in the matrix include those of the citric acid cycle. As each reaction is catalyzed by its specific enzyme, the mitochondrial matrix has a high protein density of up to 500 mg/ml, close to that in a protein crystal. For cryo-ET of intact organelles, the high matrix density has the disadvantage of obscuring internal detail.

The equivalent of the periplasm in the bacterial ancestors of mitochondria is the intermembrane space. This is the ~20 nm gap between the outer membrane and the part of the inner membrane that is known as the inner boundary membrane. All matrix proteins imported into the mitochondrion from the cytoplasm must pass through the outer and inner membrane and therefore also through the intermembrane space. Conventional EM of thin plastic sections suggested sites of direct contact between the lipid bilayers of the inner and outer membrane [24, 25], but these seem to be artifacts of fixation and dehydration. Protein translocases of the outer (TOM) and inner (TIM) membrane form a supercomplex that has been visualized by cryo-ET [26]. The TOM/TIM supercomplex spans the intermembrane space and appears to be held together by the polypeptide in transit. The inner boundary membrane must contain large numbers of the carrier proteins that shuttle ions, ATP, ADP and small metabolites between the cytoplasm and the matrix. These small membrane proteins include most notably the 33 kDa ATP/ADP carrier [27], as well as numerous other related and unrelated membrane transporters.

**Fig. 2:**
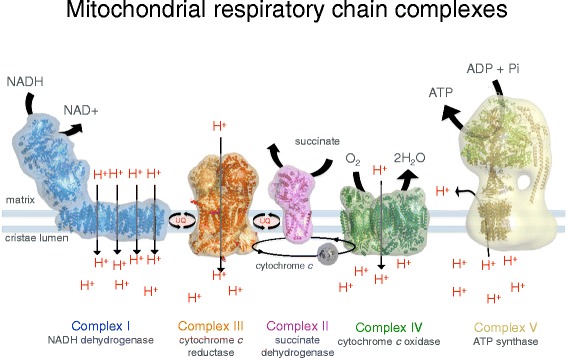
Membrane protein complexes of the respiratory chain. Electron transport complexes I (NADH/ubiquinone oxidoreductase, *blue*), II (succinate dehydrogenase, *pink*), III (cytochrome *c* reductase, *orange*), IV (cytochrome *c* oxidase, *green*) and the mitochondrial ATP synthase (also known as complex V, *tan*) work together in oxidative phosphorylation to harness energy for the cell. Complexes I, III and IV pump protons across the cristae membrane, creating the proton gradient that drives ATP synthesis. *UQ* ubiquinol. (Adapted from Davies KA, Daum B. *Biochem. Soc. Trans.* 2013;**41**:1227–34.)

The inner membrane forms invaginations, called cristae, that extend deeply into the matrix. The cristae define the third mitochondrial compartment, the crista lumen. The crista membranes contain most, if not all, of the fully assembled complexes of the electron transport chain and the ATP synthase (Fig. 2). The crista lumen contains large amounts of the small soluble electron carrier protein cytochrome *c*. The mitochondrial cristae are thus the main site of biological energy conversion in all non-photosynthetic eukaryotes.

## The inner membrane cristae

Cristae were first discovered by electron microscopy in thin sections of plastic-embedded cells and tissues [28, 29]. They are disk-like lamellar, tubular or bag-like extensions of the inner boundary membrane, and are continuous with it at the crista junctions. Crista junctions in mitochondria of post-mitotic heart or liver cells are small circular apertures of ~25 nm diameter [14, 30]. In cells that divide frequently, such as yeasts, the crista junctions tend to form 25 nm slits in the boundary membrane that are up to a few 100 nm long [26, 30]. In mitochondria of all organisms, the mitochondrial contact site and cristae organizing (MICOS) system [31], an assembly of one soluble and five membrane proteins, anchors the cristae to the outer membrane. Cristae of yeast strains where particular MICOS components have been knocked out look like concentric onion rings and have few if any junctions [32].

It is thought that the MICOS complex forms a diffusion barrier within the inner membrane at the crista junctions to account for the apparent lateral segregation of proteins between the cristae and boundary membranes. Evidence of such differences comes primarily from electron microscopy, because it has not been possible to separate cristae and boundary membranes biochemically. Immuno-staining of thin plastic sections has shown that respiratory chain complexes reside in the cristae rather than in the boundary membrane, whereas components of the protein translocases are found in the boundary and outer membranes [33]. Cryo-ET of intact mitochondria, which resolves large membrane protein complexes in situ, did not reveal any such assemblies (for example, the ATP synthase or complex I) in the boundary membrane [17], suggesting that the protein complexes of the inner membrane are indeed laterally segregated.

**Fig. 3:**
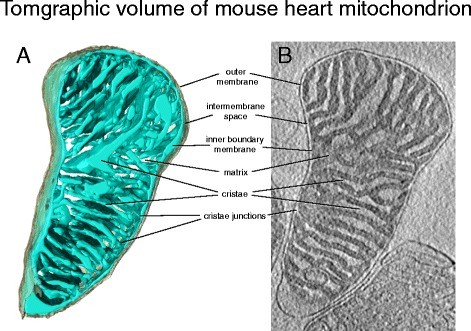
Tomographic volume of mouse heart mitochondrion. **a** Three-dimensional volume of a mouse heart mitochondrion determined by cryo-ET. The outer membrane (*grey*) envelops the inner membrane (*light blue*). The inner membrane is highly folded into lamellar cristae, which criss-cross the matrix. **b** Tomographic slice through the map volume. The dense matrix, which contains most of the mitochondrial protein, appears dark in the electron microscope, whereas the intermembrane space and crista lumen appear light because of their lower protein content. The inner boundary membrane follows the outer membrane closely at a distance of ~20 nm. The inner membrane turns sharply at the crista junctions, where the cristae join the inner boundary membrane. (Courtesy of Tobias Brandt)

In tissues with a high energy demand, such as skeletal or heart muscle, the cristae are closely stacked disk-like lamellae that take up most of the mitochondrial volume (Fig. 3). In animal tissues with lower energy demand, such as liver or kidney, the cristae are less closely stacked, leaving more room for the matrix with its biosynthetic enzymes. In all mitochondria, the cristae account for most of the inner membrane surface, highlighting their importance for cellular physiology.

## ATP synthase forms rows of dimers in crista membranes

The mitochondrial F_1_-F_o_ ATP synthase is the most conspicuous protein complex in the cristae. The ATP synthase is an ancient nanomachine that uses the electrochemical proton gradient across the inner mitochondrial membrane to produce ATP by rotatory catalysis [34]. Protons moving through the F_o_ complex in the membrane drive a rotor ring composed of 8 (in mammals [35]) or 10 (in yeast [36]) *c*-subunits. The central stalk propagates the *c*-ring torque to the catalytic F_1_ head, where ATP is generated from ADP and phosphate through a sequence of conformational changes. The peripheral stalk prevents unproductive rotation of the F_1_ head against the F_o_ complex.

**Fig. 4:**
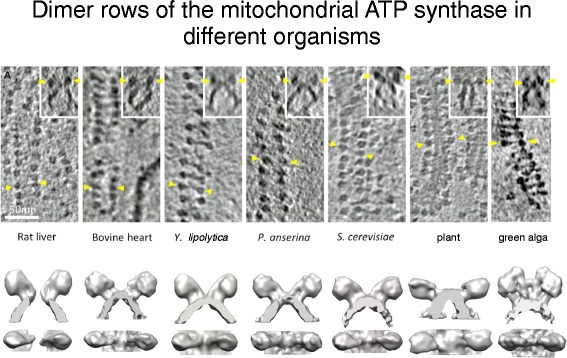
Double rows of ATP synthase in seven different species. *Top row*: slices of 3D volumes obtained by cryo-ET with rows of ATP synthase dimers. *Insets* show side views of the dimers in the membrane. *Yellow arrowheads* indicate F_1_ heads of one dimer. Scale bar = 50 nm. *Bottom row*: Surface representations of subtomogram averages. (Adapted from [17])

For many years it was assumed that the ATP synthase and other energy-converting complexes are randomly distributed over the inner membrane. The first hint that this is not the case came from deep-etch freeze-fracture electron microscopy, which revealed double rows of macromolecular complexes in the tubular cristae of the single-cell ciliate *Paramecium* [37]. The double rows were thought to be linear arrays of mitochondrial ATP synthase. This is indeed what they are, but it could only be shown unambiguously more than 20 years later by cryo-ET [30, 38], which revealed rows of ATP synthase dimers in mitochondria of all species investigated [30] (Fig. 4). Until then, the rows were thought to be peculiar to *Paramecium*.

The linear arrays of ATP synthase dimers are a ubiquitous and fundamental attribute of all mitochondria. They are always found along the most tightly curved regions along the crista ridges (Additional file 2), or around narrow tubular cristae. Subtomogram averages indicate that dimers from fungi and mammals are indistinguishable at low resolution, whereas those from plants, algae and protists differ in dimer angle or position of the peripheral stalk relative to the catalytic F_1_ head (Fig. 4). However, the basic assembly of ATP synthase complexes into dimers and their association into long rows along the crista ridges are conserved. Coarse-grained molecular dynamics simulations indicate that the dimers bend the lipid bilayer and, as a result, self-associate into rows [30]. Accordingly, row formation does not require specific protein interactions, but is driven by the energy of elastic membrane deformation. Cryo-ET of isolated, detergent-solubilized ATP synthase dimers reconstituted into proteoliposomes has provided experimental evidence that the dimers do in fact self-associate into rows and that they induce a local curvature in the lipid bilayer (Blum, Davies, Kühlbrandt, unpublished results). Therefore, it is in fact the dimers that bend the membrane, not the other way round.

**Fig. 5:**
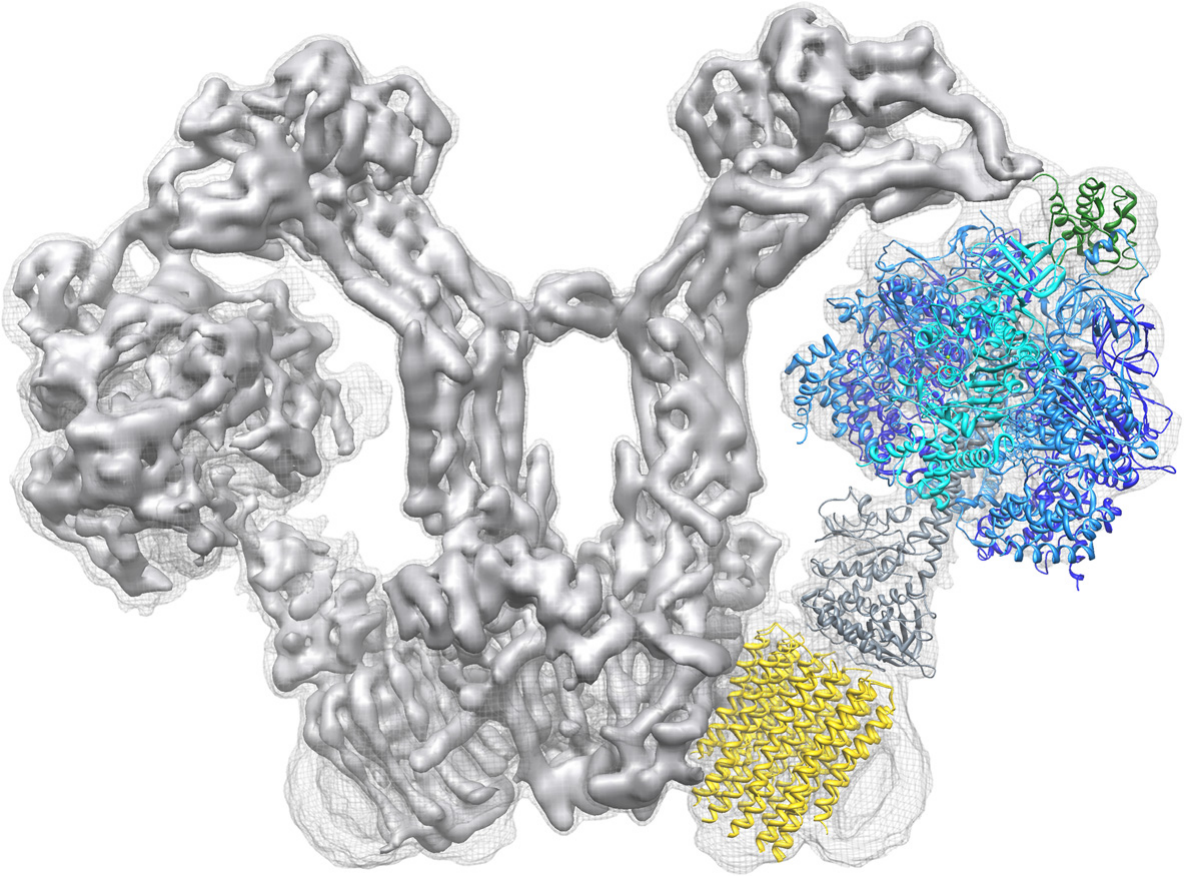
Structure of the mitochondrial ATP synthase dimer from *Polytomella* sp*.* Side view of the two mitochondrial ATP synthase in the V-shaped dimer. One protomer is fitted with atomic models PDB 2WSS [63] (α-subunits, *cyan*; β-subunits, *blue*; γδε subunits, *grey*; OSCP, *green*) and PDB 3U2Y [64] (*yellow*, *c*_10_-ring). Density threshold levels are 1σ (*mesh*) or 7σ (*solid surface*)*.* (Adapted from [40])

The structure of a mitochondrial ATP synthase dimer has now been determined by single-particle cryo-EM at 6–7 Å resolution [39]. Surprisingly, the structure revealed a hairpin of long, membrane-intrinsic α-helices in the *a*-subunit next to the F_o_ rotor ring, as well as aqueous half-channels on either side of the membrane for proton translocation. The long helices appear to play a central role in this process, as they are preserved in all rotary ATPases [40]. Together with the fitted high-resolution X-ray structures of the catalytic F_1_ head and the rotor ring in the membrane, the cryo-EM map provides the first complete picture of this pivotal mitochondrial membrane complex (Fig. 5) and goes a long way towards explaining its mechanism [40].

The ubiquitous nature of the dimer rows raises the question as to the biological significance of this striking, conserved arrangement. In yeast the two protomers are linked by the dimer-specific ATP synthase subunits *e* and *g*. If either subunit is knocked out, only monomeric ATP synthase is found in the inner membrane [30] and the usual lamellar or tubular cristae do not form [30, 41]. ATP synthase dimers and dimer rows are thus a prerequisite for proper cristae formation. Although the loss of the dimer-specific subunits is not lethal, it is a serious disadvantage. Yeast subunit *e* and *g* knockout strains grow up to 60 % more slowly than wild type and their mitochondria only have about half the membrane potential [42]. This raises the further question about the role of the cristae, and hence the dimer rows, in cellular physiology and fitness. Most likely the invaginations prevent protons that are pumped into the crista lumen by the respiratory chain from escaping rapidly to the inter-membrane space and the cytoplasm, so that they can be harnessed more efficiently for ATP production. In this way, the cristae, and hence the dimer rows, would contribute to effective ATP synthesis.

Prokaryotic ATP synthases lack the dimer-specific subunits, and ATP synthase dimers or dimer rows have not been found in bacterial or archaeal inner membranes, which also do not have cristae. The cristae and dimer rows may thus be an adaptation that enables mitochondria to satisfy the high energy demand of eukaryotic cells with the available, shallow proton gradient of around 0.6–0.8 pH units. ATP synthase dimers have recently been implicated in the formation of the permeability transition pore [43] that triggers apoptosis. On the basis of the structure of the mitochondrial ATP synthase dimer [39] or the dimer rows [30], however, it is difficult to see how they might form a membrane pore.

## Respiratory chain complexes and supercomplexes

The proton gradient across the cristae membrane is generated by three large membrane protein complexes of the respiratory chain in the cristae, known as complex I (NADH/ubiquinone oxidoreductase), III (cytochrome *c* reductase) and IV (cytochrome *c* oxidase) (Fig. 2). Complex I feeds electrons from the soluble carrier molecule NADH into the respiratory chain and transfers them to a quinol in the membrane. The energy released in the electron transfer reaction is utilized for pumping four protons from the matrix into the crista lumen. Complex III takes the electrons from the reduced quinol and transfers them to the small, soluble electron carrier protein cytochrome *c*, pumping one proton in the process. Finally, complex IV transfers the electrons from cytochrome *c* to molecular oxygen and contributes to the proton gradient by using up four protons per consumed oxygen molecule to make water. Complex II (succinate dehydrogenase) transfers electrons from succinate directly to quinol and does not contribute to the proton gradient.

**Fig. 6:**
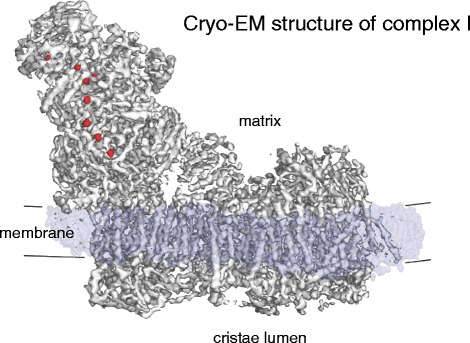
Cryo-EM structure of bovine heart complex I. Mitochondrial complex I (~1 MDa) has a matrix arm and a membrane arm. The matrix arm contains a row of eight iron-sulfur clusters (*red*) that conduct electrons from NADH to ubiquinol at the junction of the matrix and membrane arms (Fig. 7). The membrane arm consists of 78 trans-membrane helices, including three proton-pumping modules. (Adapted from [51]; EMDB code 2676)

The respiratory chain complexes have been studied in great detail for decades. High-resolution X-ray structures are available for mitochondrial complex III [44] and IV [45]. At a molecular mass of ~1 megadalton (MDa), mitochondrial complex I is far larger and has more subunits than complexes III and IV put together. As yet there is no X-ray structure of the mammalian complex, but very recently a ~3.6 Å X-ray structure of complex I from the obligate aerobic yeast *Yarrowia lipolytica* has been obtained [46]. Comparison to the high-resolution X-ray structure of the ~550 kDa complex I from the thermophilic bacterium *Thermus thermophilus* [47] indicates that the 14 conserved core subunits have essentially the same structure in both, including three proton antiporter modules in the membrane and eight iron-sulfur clusters in the matrix arm. The mitochondrial complex has about three times as many protein subunits as its bacterial ancestor. Most functions of the extra subunits are unknown, but many of them are likely to work in assembly or the regulation of complex I function. Features that are conserved from bacteria to mitochondria include a long horizontal α-helix on the matrix side that may stabilize the membrane domain. The recent 5 Å single-particle cryo-EM structure of bovine heart complex I (Fig. 6) has resolved the proton-translocating modules, iron-sulfur clusters and long horizontal helix, and 14 of the 31 supernumerary mammalian complex I subunits have been identified [48]. However, the way in which electron transfer from NADH to ubiquinone in complex I is coupled to proton translocation is still unknown, and much else remains to be discovered.

**Fig. 7:**
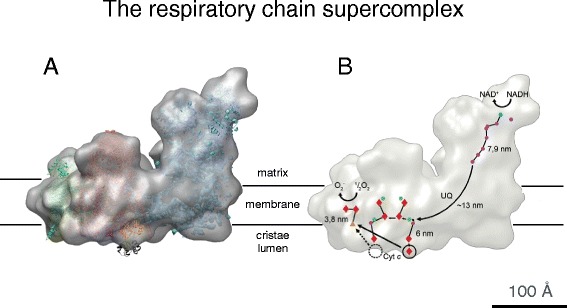
Cryo-EM structure of the 1.7 MDa bovine heart respiratory chain supercomplex. **a** The supercomplex consists of one copy of NADH dehydrogenase (complex I, *blue*), a cytochrome *b-c*_*1*_ dimer (complex III, *pink*), and a single copy of cytochrome *c* oxidase (complex IV, *green*). **b** The ubiquinol (*UQ*) binding sites of complexes I and III and the short distance between the cytochrome *c* binding sites in complexes III and IV, which would favor efficient electron transfer. Cofactors active in electron transport are marked in *yellow* (FMN), *orange* (iron–sulfur clusters), *dark blue* (quinols), *red* (hemes), and *green* (copper atoms). *Arrows* indicate the electron path through the supercomplex. (Adapted from [51])

**Fig. 8:**
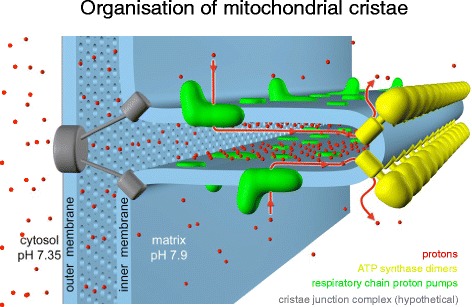
ATP synthase dimer rows shape the mitochondrial cristae. At the cristae ridges, the ATP synthases (*yellow*) form a sink for protons (*red*), while the proton pumps of the electron transport chain (*green*) are located in the membrane regions on either side of the dimer rows. Guiding the protons from their source to the proton sink at the ATP synthase, the cristae may work as proton conduits that enable efficient ATP production with the shallow pH gradient between cytosol and matrix. *Red arrows* show the direction of the proton flow. (Adapted from [17])

Not unlike the ATP synthase, which forms dimer rows in the cristae, the proton pumps of the electron transport chain assemble into supercomplexes or ‘respirasomes’. Respiratory chain supercomplexes were first postulated on the basis of blue-native gels of yeast and bovine heart mitochondria solubilized in the mild detergent digitonin [49]. Negative-stain electron microscopy [50] and single-particle cryo-EM [51] of the 1.7 MDa bovine heart supercomplex revealed that it consists of one copy of complex I, one complex III dimer, and one complex IV monomer. X-ray structures of the component complexes were fitted to the 3D map (Fig. 7) [51], indicating the path of the electron from NADH via the iron-sulfur clusters of complex I and ubiquinol to the prosthetic groups of complex III, and finally to molecular oxygen in complex IV. Genetic evidence provides strong support for the existence of respirasomes in vivo [52], but they were long thought to be artifacts of detergent solubilization, notwithstanding their well defined structure. Recent cryo-ET work has shown that they do exist in cristae membranes of bovine heart mitochondria (Davies and Kühlbrandt, unpublished results). *Saccharomyces cerevisiae*, which lacks complex I, nevertheless has a respiratory chain supercomplex consisting of complex III and IV [53]. Far from being randomly distributed in the membrane, the ATP synthase and electron transport complexes of the respiratory chain thus form supramolecular assemblies in the cristae, in a way that is essentially conserved from yeast to humans (Fig. 8). A clear functional role of mitochondrial supercomplexes has not yet been established. They may make electron transfer to and from ubiquinone in complexes I and III more efficient, as the relative positions and orientations of the two complexes are precisely aligned rather than random. However, there is no direct evidence that this makes a difference. The supercomplexes may simply help to avoid random, unfavorable protein–protein interactions in the packed environment of the inner mitochondrial membrane [54]. Alternatively, they may control the ratio of respiratory chain complexes in the membrane, or aid their long-term stability.

A main protein component of the crista lumen is the small soluble electron carrier protein cytochrome *c* that shuttles electrons from complex III to complex IV. If released into the cytoplasm, cytochrome *c* triggers apoptosis [55]. It is imperative, therefore, that cytochrome *c* does not leak from the cristae and that the outer membrane remains tightly sealed during mitochondrial fission and fusion.

## Membrane rearrangement during cellular ageing

**Fig. 9:**
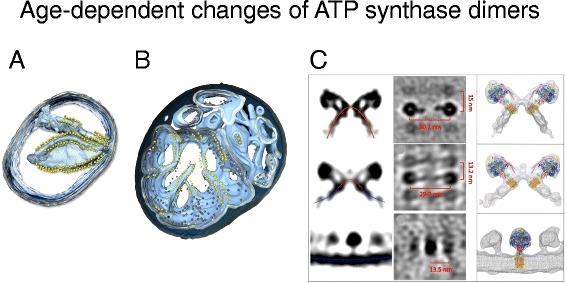
Changes of inner membrane morphology and ATP synthase dimers in ageing mitochondria. Tomographic volumes of mitochondria isolated from young (6-day-old) (**a**) and ageing (13-day-old) (**b**) cultures of the model organism *Podospora anserina*. In young mitochondria, the ATP synthase dimers are arranged in rows along highly curved inner membrane ridges (Movie S2). In ageing mitochondria, the cristae recede into the boundary membrane, with ATP synthases dimer rows along the shallow inner membrane ridges. Outer membrane, *transparent grey*; inner membrane, *light blue*. ATP synthase F_1_ heads are shown as *yellow spheres*. **c** Side and top views (left and centre) of subtomogram-averaged ATP synthase dimers from 6-day-old (*top row*), 9-day-old (*centre*), and 15-day-old *P. anserina* mitochondria. *Right*: subtomogram averages with fitted X-ray models. *Red lines*, convex membrane curvature (as seen from the matrix); *blue lines*, concave membrane curvature. (Adapted from [56])

Ageing is a fundamental yet poorly understood biological process that affects all eukaryotic life. Deterioration in mitochondria is clearly seen in ageing, but details of the underlying molecular events are largely unknown. Cryo-ET of mitochondria from the short-lived (~18 days) model organism *Podospora anserina* revealed profound age-dependent changes in the inner membrane architecture [56]. In normal mitochondria of young cells, the cristae protrude deeply into the matrix. Formation of cristae depends both on the rows of ATP synthase dimers along the edges [30] and on the MICOS complex at the crista junctions [32]. With increasing age, the cristae recede into the inner boundary membrane and the inter-membrane space widens. The MICOS complex most likely has to come apart for this to happen. Eventually, the matrix breaks up into spherical vesicles within the outer membrane. The ATP synthase dimer rows disperse and the dimers dissociate into monomers. As the inner membrane vesiculates, the sharp local curvature at the dimer rows inverts, so that the ATP synthase monomers are surrounded by a shallow concave membrane environment, rather than the sharply convex curvature at the crista ridges (Fig. 9). Finally, the outer membrane ruptures, releasing the inner membrane vesicles, along with apoptogenic cytochrome *c*, into the cytoplasm. Cytochrome *c* activates a cascade of proteolytic caspases, which degrade cellular proteins [55]. The cell enters into apoptosis and dies.

The observed morphological changes during ageing in *P. anserina* mitochondria may be a direct or indirect consequence of cumulative oxidative damage, which is known to promote cellular and organismal senescence [57]. The electron-transfer reactions in complexes I and III generate reactive superoxide radicals as side products [58], which cause damage to mitochondrial proteins and DNA, as well as to other cellular components. Senescent mitochondria that lack cristae and ATP synthase dimers would not be able to provide sufficient ATP to maintain essential cellular functions. Cells normally deal with oxidative damage by oxygen radical scavenging enzymes such as superoxide dismutase or catalase, as well as by mitochondrial fission and fusion. Damaged or dysfunctional mitochondria are either complemented with an undamaged part of the mitochondrial network by fusion or sorted out for mitophagy [59]. During ageing, fission overpowers fusion and the mitochondrial network fragments [60]. This prevents the complementation of damaged mitochondria by fusion and thus accelerates their deterioration.

## Open questions

Even though mitochondria and their membrane protein complexes have been studied intensely for more than five decades, they remain a constant source of fascinating and unexpected new insights. Open questions abound, many of them of a fundamental nature and of direct relevance to human health [61].

Concerning macromolecular structure and function, we do not yet understand the precise role of the highly conserved feature of ATP synthase dimers and dimer rows in the cristae and the interplay between the MICOS complex and the dimer rows in cristae formation. Are there other factors involved in determining crista size and shape?

We still do not know how complex I works, especially how electron transfer is coupled to proton translocation. What is the role of respiratory chain supercomplexes? Do they help to prevent oxidative damage to mitochondria, and if so, how? And how does this affect ageing and senescence?

We also do not know how the TIM and TOM protein translocases work, and what they look like at high resolution. The same is true for the structure of the MICOS complex at the crista junctions. How does it anchor the cristae to the outer membrane, and how does it separate the cristae form the contiguous boundary membrane? Similarly, the mechanisms of mitochondrial fission and fusion and the precise involvement and coordination of the various protein complexes in this intricate process is a fascinating area of discovery.

The biogenesis and assembly of large membrane protein complexes in mitochondria is largely unexplored. Where and exactly how do the respiratory chain complexes and the ATP synthase assemble? How is their assembly from mitochondrial and nuclear gene products coordinated? Does this involve feedback from the mitochondrion to the cytoplasm or the nucleus, and what is it?

And finally, how exactly are mitochondria implicated in ageing? Why do some cells and organisms live only for days, while others have lifespans of years or decades? Is this genetically programmed or simply a consequence of different levels of oxidative damage? How is this damage prevented or controlled, and how does it affect the function of mitochondrial complexes? Is the breakdown of ATP synthase dimers also an effect of oxidative damage, and is it a cause of ageing?

It will be challenging to find answers to these questions because many of the protein complexes involved are sparse, fragile and dynamic, and they do not lend themselves easily to well established methods, such as protein crystallography. Cryo-EM, which is currently undergoing rapid development in terms of high-resolution detail, will have a major impact but is limited to molecules above about 100 kDa [62]. Even better, more sensitive electron detectors than the ones that have precipitated the recent resolution revolution, in combination with innovative image processing software, will yield more structures at higher resolution. However, small, rare and dynamic complexes will remain difficult to deal with. New labeling strategies in combination with other biophysical and genetic techniques are needed. Cloneable labels for electron microscopy, equivalent to green fluorescent protein in fluorescence microscopy, would be a great help; first steps in this direction look promising [26]. Once the structures and locations of the participating complexes have been determined, molecular dynamics simulations, which can analyze increasingly large systems, can help to understand their molecular mechanisms. Without any doubt, mitochondria and their membrane protein complexes will remain an attractive research area in biology for many years to come.

## Additional files

Additional file 1:
**Movie S1.** Mitochondria in a human endothelial cell. Time-lapse movie of the dynamic mitochondrial network stained with a fluorescent dye. Long filamentous mitochondria occasionally undergo fission, while smaller parts of the network fuse into longer tubes. (Courtesy of Jürgen Bereiter-Hahn, Goethe University, Frankfurt, Germany) (AVI 137694 kb) Additional file 2:
**Movie S2.** Dimer rows of mitochondrial ATP synthase in cristae membranes. The three-dimensional volume of a small *P. anserina* mitochondrion obtained by cryo-ET shows that ATP synthase dimers form long rows along cristae ridges. The outer membrane is *grey*, the inner membrane and cristae membranes are *light blue*. The F_1_ heads of the ATP synthase are indicated in *yellow*. (Adapted from [17]) (MP4 7740 kb)
